# Shared decision-making in psoriasis care: Evaluation of how patients’ perception of clinicians’ delivery of care changes by age and sex

**DOI:** 10.1371/journal.pone.0303058

**Published:** 2024-05-10

**Authors:** Robin Kikuchi, Paige Kingston, Audrey Hao, Kaviyon Sadrolashrafi, Rebecca K. Yamamoto, Hannah Tolson, Sara N. Bilimoria, Lily Guo, Danielle Yee, Maria T. Ochoa, April W. Armstrong

**Affiliations:** 1 Keck School of Medicine, University of Southern California, Los Angeles, California, United States of America; 2 Kirk Kerkorian School of Medicine at UNLV, Las Vegas, Nevada, United States of America; 3 Georgetown University School of Medicine, Washington, DC, United States of America; 4 University of Arizona College of Medicine, Phoenix, Arizona, United States of America; 5 Division of Dermatology, Department of Medicine, David Geffen School of Medicine, University of California Los Angeles, Los Angeles, California, United States of America; 6 Duke University School of Medicine, Durham, North Carolina, United States of America; 7 Department of Dermatology, Keck School of Medicine, University of Southern California, Los Angeles, California, United States of America; Pennsylvania State University Hershey Medical Center, UNITED STATES

## Abstract

**Background:**

Shared decision-making (SDM) refers to a collaborative process in which clinicians assist patients in making medically informed, evidence-based decisions that align with their values and preferences. There is a paucity of literature on SDM in dermatology.

**Objective:**

We aim to assess whether male and female psoriasis patients evaluate their clinicians’ engagement in SDM differently across different age groups.

**Methods:**

Cross-sectional study using data from the 2014–2017 and 2019 Medical Expenditure Panel Surveys (MEPS).

**Results:**

A weighted total of 7,795,608 psoriasis patients were identified. SDM Scores ranged from 1 to 4, with 4 representing the most favorable patient evaluation of their clinicians’ engagement in SDM. We conducted multivariate linear regression to compare mean SDM Scores in male psoriasis patients versus female psoriasis patients across different patient age groups. Female patients ages 60–69 perceived significantly greater clinician engagement in SDM compared to age-matched male patients (female patient perception of SDM **3.65** [95%CI:3.61–3.69] vs. male patient perception of SDM **3.50** [95%CI:3.43–3.58], p<0.005). The same trend of older female patients evaluating their clinicians’ engagement in SDM significantly higher than their age-matched male counterparts exists for the age group >70 (p<0.005). No significant differences between male and female patients’ evaluations of their clinicians’ engagement in SDM were demonstrated in subjects younger than 60. All calculations were adjusted for demographic and clinical factors.

**Conclusions:**

Compared to older male psoriasis patients, older female psoriasis patients evaluated their clinicians to be more engaged in shared decision-making.

## Introduction

Psoriasis is an immune-mediated dermatological disorder affecting 3% of the United States (US) population [1]. Current literature reports that greater than 50% of psoriasis patients are dissatisfied with their care [2] and 40% are non-adherent with their prescribed regimens [3]. Poor communication between physicians and patients may contribute to this non-adherence. Therefore, building strong patient-physician relationships is essential to improve patient adherence to and satisfaction with treatment interventions [4–7].

Shared decision-making (SDM) describes a method of delivering healthcare in which clinicians assist patients in making clinically informed, evidence-based decisions based on the values, needs, and circumstances that affect patient care [4, 8]. The Institute of Medicine’s Committee on Improving the Quality of Cancer Care cites that SDM can be accomplished when clinicians actively listen to patients, respectfully communicate with patients, educate patients using easily understood language, include patients in the decision-making process, and ensure patients have the ability to adhere to proposed regimens [4, 5, 9].

A recent study querying a nationally representative database demonstrated a significant positive association between psoriasis patients’ satisfaction with care and their perceptions of clinicians’ delivery of SDM. However, only 42% of the cohort reported engaging in high-quality SDM [10]. Few studies have examined whether psoriasis patients’ demographic factors affect their participation in SDM. However, studies in the general medical setting have found that male patients demonstrate decreased participation in SDM as compared to female patients [11]. In addition, elderly US patients report physicians’ non-responsiveness to their concerns as the leading factor discouraging them from seeking medical care [12]. However, the interaction between a patient’s age and sex in SDM is not well understood. For example, it is unknown whether there are different experiences with SDM between older male and older female patients and younger male and younger female patients or between any combination of these patient groups.

Determining specific demographic factors that impact psoriasis patients’ perceptions of SDM may improve our understanding of satisfaction with and adherence to treatments. In this study, we aim to assess whether male and female psoriasis patients evaluate their clinicians’ engagement in SDM differently across different age groups.

## Materials and methods

### Data source and study population

This study was conducted using data collected by the Medical Expenditure Panel Survey (MEPS), a cross-sectional survey of US patients. The MEPS is the largest publicly available database collecting data on healthcare utilization and expenditure as well as participant demographics and comorbidities through large-scale surveys. They collect de-identified data from households and individuals across the US that is supplemented by participants’ medical providers and employers [13]. As we utilized publicly available, de-identified data for this study, it was exempt from Institutional Review Board approval.

Survey responses were included for respondents ≥18 years old with a diagnosis of psoriasis during the years 2013–2017 and 2019. The year 2018 was excluded, as the variables used to assess SDM were not collected during that year. We used the MEPS Medical Conditions data file to identify psoriasis patients using the International Classification of Diseases, Ninth Revision (ICD-9) code 696 or the International Statistical Classification of Diseases and Related Health Problems, Tenth Revision (ICD-10) code L40. We used the MEPS Full-Year Consolidated File and Prescribed Medicines File to obtain the study population’s sociodemographic characteristics and responses to the variables used to assess SDM.

### Variables

The independent variable of patients’ ages was collected and categorized into age groups: <30, 30–39, 40–49, 50–59, 60–69, and >70 years old. The independent variable for patient sex was collected from self-reported categories of male and female.

The dependent variable was the composite SDM Score, representing patients’ evaluation of clinicians’ engagement in SDM. The SDM Score was based on methodologies from prior research using the MEPS.

The SDM Score was based on methodologies from prior research using the MEPS. The score was developed by Fiks et al. in the primary care setting and is composed of seven variables (Table 1), based on the most widely accepted definition of SDM [9, 14]. The SDM Score has been used with strong predictive value in the dermatologic setting [10, 15]. First, a cross-sectional study by Yee et al. used the SDM Score to assess psoriasis patients’ perceptions of clinicians’ delivery of SDM, demonstrating an unfavorable perception across the population [10]. Additionally, another cross-sectional study by Kingston et al. utilized the SDM Score to evaluate patient perceptions’ of SDM in acne care between patients with skin of color and white patients. Acne patients with skin of color were significantly more likely to engage in high levels of SDM as compared to their white counterparts [15]. The SDM Score has not been used in other dermatologic diseases. Of the seven variables that compose the SDM score, six were coded between 1 and 4, where 1 is “never,” 2 is “sometimes,” 3 is “usually,” and 4 is “always.” One variable collected a binary response, and the variable was coded for a score of 1 being “no” and 4 being “yes” for numerical consistency. The SDM Score is the mean response to all seven items and ranges from 1 to 4. An SDM Score of 4 represents the most favorable patient rating of clinicians’ engagement in SDM. Using latent class analysis, prior literature has established a score of 3.8 or lower to represent an unfavorable evaluation [14, 16].

**Table 1:** Variables in the MEPS used to assess SDM in psoriasis patients.

Variable	Question
**Decision Score**	If there were a choice between treatments, how often would your medical provider ask you to help make the decision?
**History Taking Score**	Does someone at your usual provider usually ask about prescription medications and treatments other doctors may give?
**Listen Score**	How often did doctors or other health providers listen carefully to you?
**Patient Education Score**	How often did doctors or other health providers explain things in a way that was easy to understand?
**Respect Score**	How often did doctors or other health providers show respect for what you had to say?
**Time Spent Score**	How often did doctors or other health providers spend enough time with you?
**Option Score**	Does a medical person at your usual source of care present and explain all options to you?

### Covariates

Demographic covariates included self-reported sex, race, ethnicity, marital status, household income, educational attainment, employment status, and healthcare insurance coverage. Clinical covariates included psoriasis treatment type (no treatment, topicals, oral systemics, and biologics), perceived health status, and Charlson Comorbidity Index (CCI).

### Analytical approach: Sampling weights, multivariate regression

We performed a descriptive analysis of the demographic and clinical variables for the weighted cohort. Based on the recommendation of the MEPS, we applied person-level sampling weights to reflect the sampling method used in the survey administration methodology [13].

We performed multivariate linear regression to compare patients’ evaluation of clinicians’ engagement in SDM, represented by mean SDM Scores, in male psoriasis patients versus female psoriasis patients across different patient age groups. We adjusted the regression model for psoriasis disease severity using psoriasis treatment type (none vs. topical vs. systemic), comorbidities as measured by Charlson Comorbidity Index, socioeconomic status as measured by household income and employment status, and educational level as measured by educational attainment. Additionally, we controlled for insurance coverage status, race, ethnicity, marital status, and perceived health status. We conducted the same analysis for each of the seven variables contributing to the composite SDM Score.

We also performed multivariate linear regression to determine the relationship between patients’ evaluation of clinicians’ engagement in SDM, represented by mean SDM Score, and patient age. We adjusted the regression model for potentially confounding variables as above. A statistically significant result was defined as a p-value less than 0.05. All statistical analyses were performed using STATA (StataCorp LP, College Station,TX).

## Results

### Demographics

A weighted total of 7,795,608 patients were included in the analysis. The demographic characteristics are summarized in Table 2. The average composite SDM Score was 3.60 out of 4.

**Table 2:** Weighted sample demographic and clinical characteristics for psoriasis patients.

	Cohort (N = 7,795,608)
Variables	N (%)
**Mean Shared Decision-Making (SEM)**	3.60 (0.02)
**Mean Age, years (SEM)**	56.12 (0.64)
**Self-Reported Sex**	
**Male**	2,642,711 (33.90%)
**Female**	5,152,897 (66.10%)
**Self-Reported Race**	
**White**	6,482,048 (83.15%)
**Black**	525,424 (6.74%)
**American Indian/Alaska Native**	73,279 (0.94%)
**Asian American/Native Hawaiian/Pacific Islander**	277,524 (3.56%)
**Multiple**	438,113 (5.62%)
**Ethnicity**	
**Hispanic**	1,182,594 (15.17%)
**Non-Hispanic**	6,613,014 (84.83%)
**Educational Attainment**	
**No Degree**	480,209 (6.16%)
**High School Degree/GED**	3,036,389 (38.95%)
**HS Degree**	3,342,757 (42.88%)
**Bachelors/Advanced**	934,693 (11.99%)
**Employment Status**	
**Employed**	4,685,940 (60.11%)
**Unemployed**	3,209,668 (39.89%)
**Marital Status**	
**Married**	4,671,908 (59.93%)
**Unmarried**	3,123,700 (40.07%)
**Insurance Status**	
**Private**	6,116,434 (78.46%)
**Public**	1,518,584 (19.48%)
**Uninsured**	160,590 (2.06%)
**Income**	
**Very Low Income**	715,637 (9.18%)
**Low Income**	802,948 (10.30%)
**Middle Income**	2,174,975 (27.90%)
**High Income**	4,102,049 (52.62%)
**Charlson Comorbidity Index (CCI), mean (SEM)**	0.17 (0.02)
**Perceived Health Status**	
**Excellent**	919,882 (11.80%)
**Very Good**	2,437,687 (31.27%)
**Good**	2,919,455 (37.45%)
**Fair**	1,299,528 (16.67%)
**Poor**	218,057 (2.81%)
**Treatment for Psoriasis**	
**No Treatment**	3,985,115 (51.12%)
**Topicals**	2,160,943 (27.72%)
**Oral Systemics**	1,152,970 (14.79%)
**Biologics**	496,580 (6.37%)

SEM = Standard Error of the Mean; GED = General Education Development.

### Analysis of composite SDM Score by age and sex

**Fig 1:**
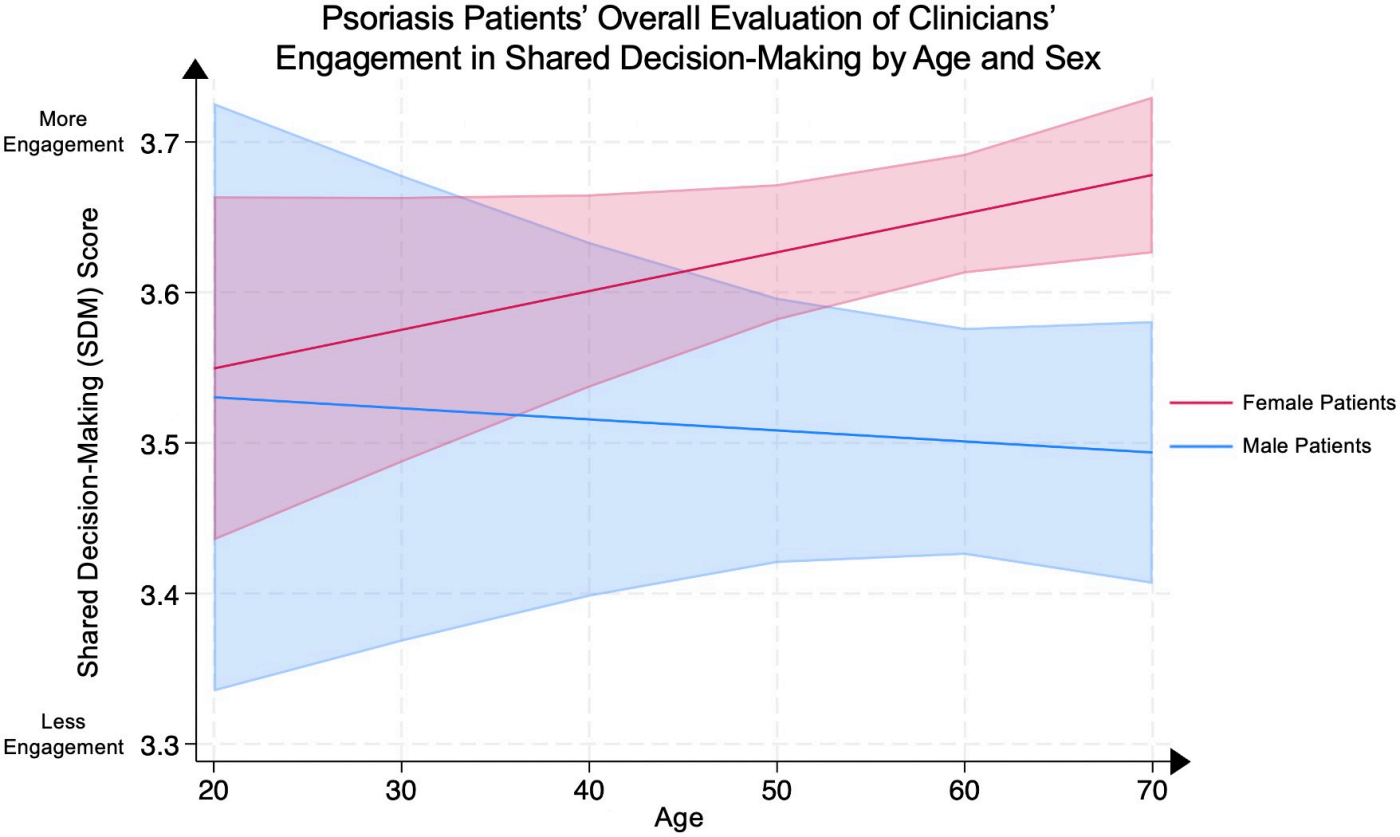
Psoriasis patients’ overall evaluation of clinicians’ engagement in shared decision-making by age and sex. Mean composite shared decision-making score, representing patients’ overall evaluation of their clinicians’ engagement in shared decision-making, in male and female psoriasis patients by their age groups. Scores range from 1 through 4, with 4 representing the most favorable patient rating. The line represents the mean score, and the shaded areas represent the 95% Confidence Intervals.

We conducted a multivariate linear regression between patients’ evaluation of clinicians’ engagement in SDM and patient age in female psoriasis patients as compared to male psoriasis patients, adjusted for covariates. As compared to age-matched male patients, older female patients demonstrated significantly higher SDM scores at ages 60–69 (female patient mean **3.65** [95% CI: 3.61–3.69] vs. male patient mean **3.50** [95% CI: 3.43–3.58], p<0.005) and >70 (female patient mean **3.68** [95% CI: 3.63–3.73] vs. male patient mean **3.49** [95% CI: 3.41–3.58], p<0.005). No significant differences were demonstrated in other age groups between male and female psoriasis patients (Fig 1).

### Analysis of decision score by age and sex

**Fig 2:**
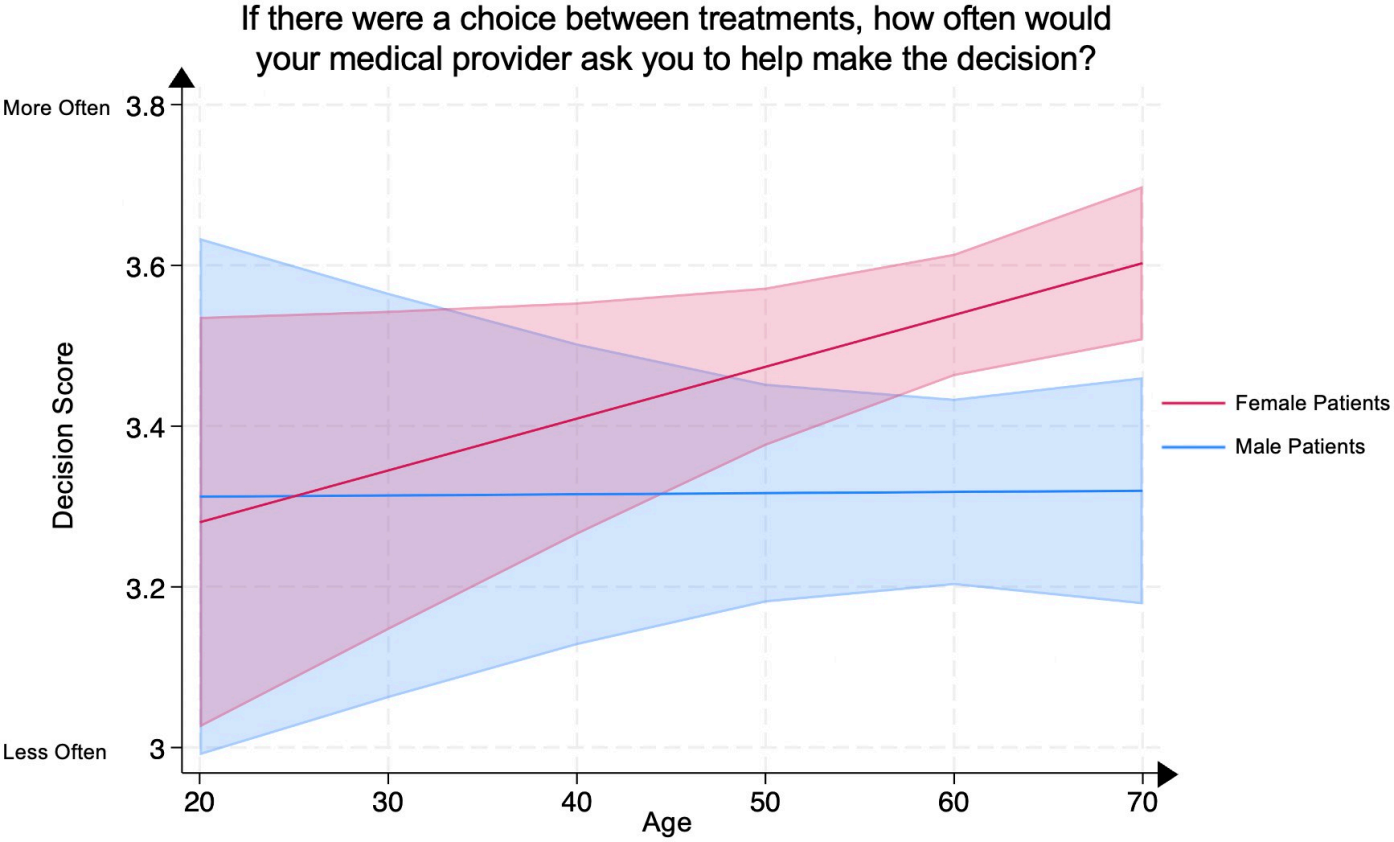
Mean decision score in male and female psoriasis patients by age group. The Decision Score represents the question “If there were a choice between treatments, how often would your medical provider ask you to help make the decision?” Scores range from 1 through 4, with 4 representing the most favorable patient rating. The line represents the mean score, and the shaded areas represent the 95% Confidence Intervals.

We conducted a multivariate linear regression between the Decision Score, representing patients’ evaluation of their inclusion in the decision-making process, and patient age in female psoriasis patients compared to male psoriasis patients, adjusting for covariates. Compared to age-matched male psoriasis patients, older female psoriasis patients demonstrated significantly higher Decision Scores at ages 60–69 (female patient mean **3.54** [95% CI: 3.46–3.61] vs. male patient mean **3.32** [95% CI: 3.20–3.43], p<0.005) and >70 (female patient mean **3.60** [95% CI: 3.51–3.70] vs. male patient mean **3.32** [95% CI: 3.18–3.46], p<0.005). No significant differences were demonstrated in other age groups between male and female psoriasis patients (Fig 2).

### Analysis of history taking score by age and sex

**Fig 3:**
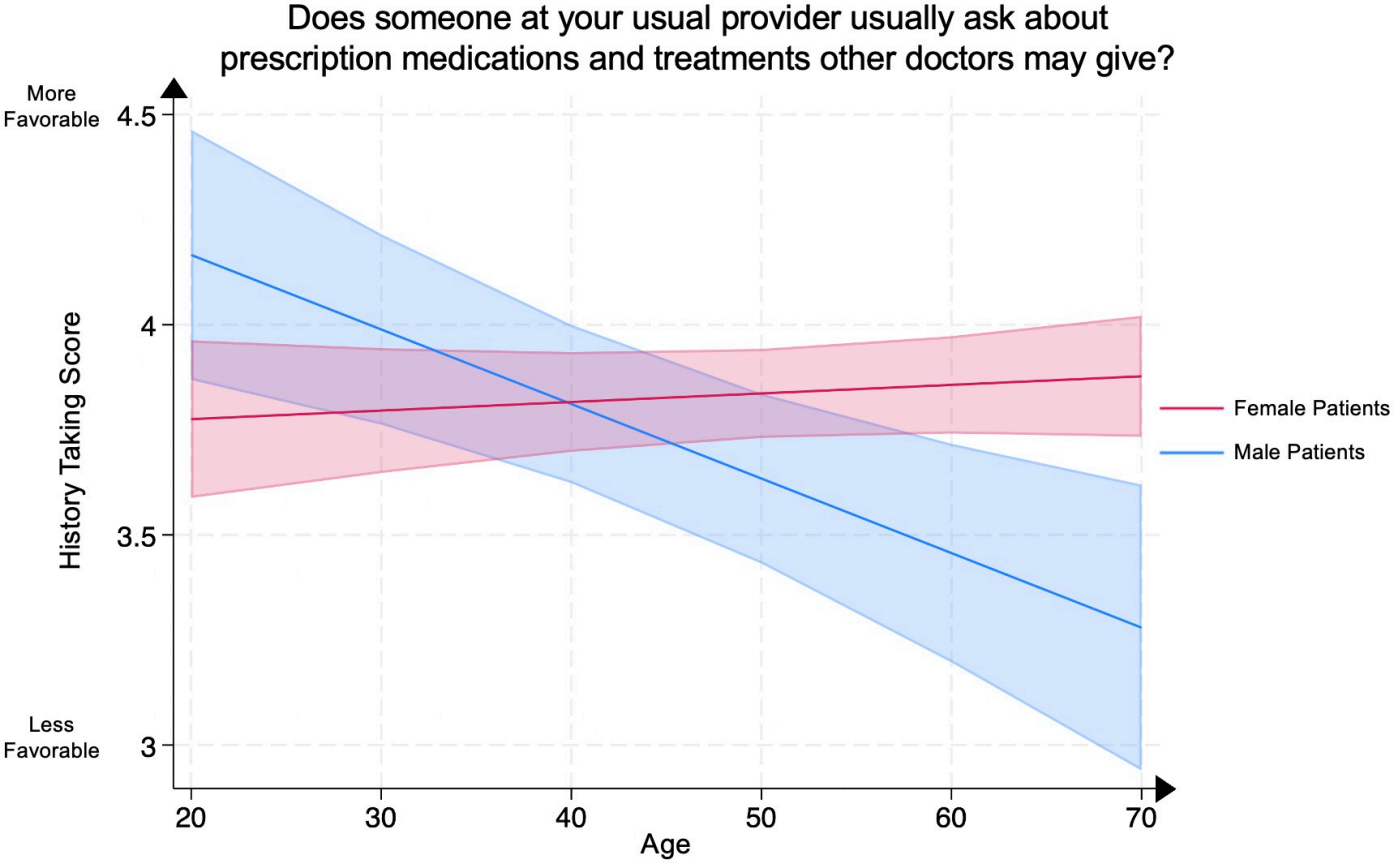
Mean history taking score in male and female psoriasis patients by age group. The History Taking Score represents the question “Does someone at your usual provider usually ask about prescription medications and treatments other doctors may give?” Scores range from 1 through 4, with 4 representing the most favorable patient rating. The line represents the mean score, and the shaded areas represent the 95% Confidence Intervals.

We conducted a multivariate linear regression between History Taking Score, representing patients’ evaluation of their clinicians’ assessment of their medical treatment history, and patient age in female psoriasis patients compared to male psoriasis patients, adjusting for covariates. Compared to age-matched male psoriasis patients, older female psoriasis patients demonstrated significantly higher History Taking Scores at ages 60–69 (female patient mean **3.86** [95% CI: 3.74–3.97] vs. male patient mean **3.46** [95% CI: 3.20–3.72], p<0.005) and >70 (female patient mean **3.88** [95% CI: 3.73–4.02] vs. male patient mean **3.28** [95% CI: 2.94–3.62], p<0.005). No significant differences were demonstrated in other age groups between male and female psoriasis patients (Fig 3).

### Analysis of option score by age and sex

**Fig 4:**
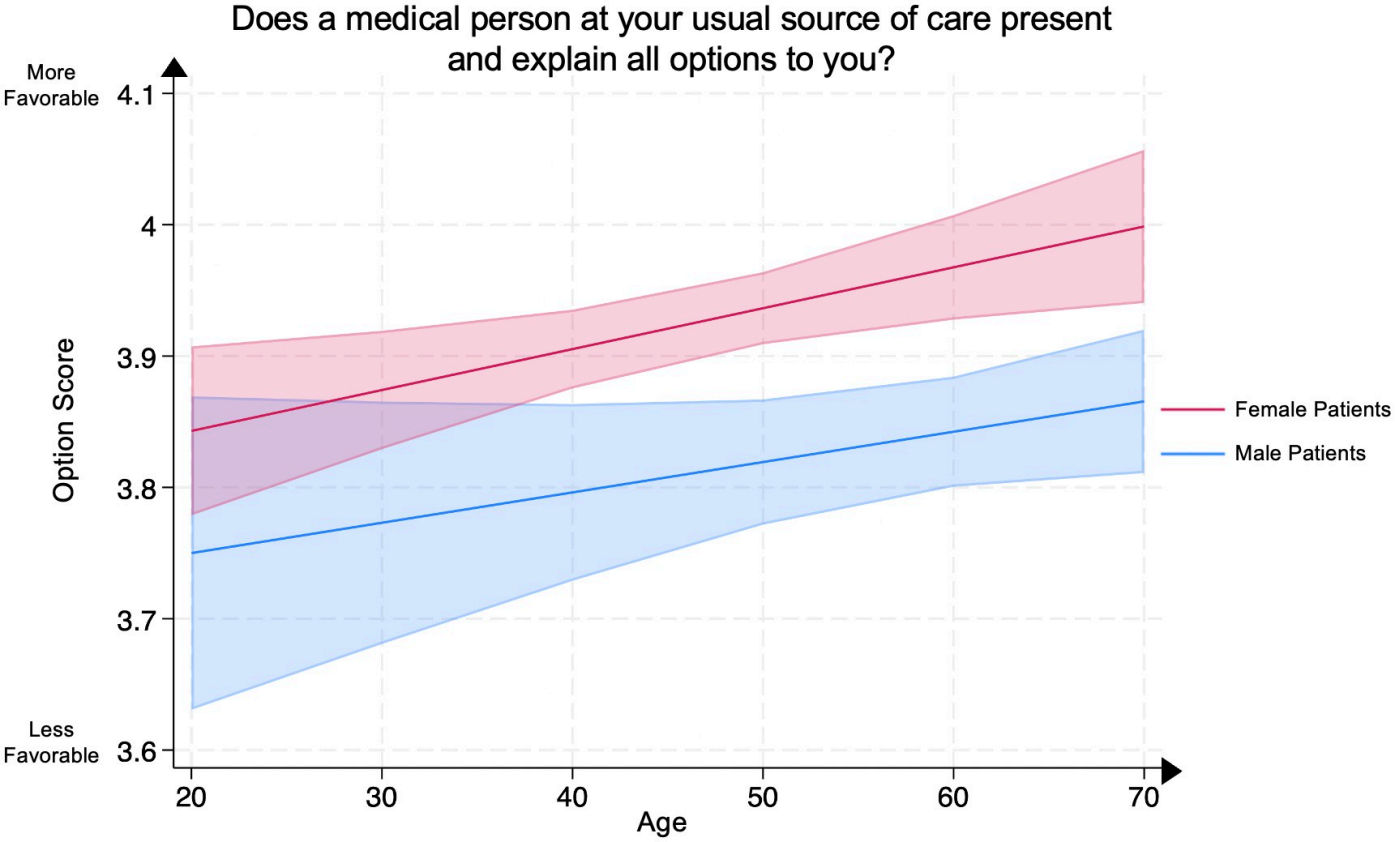
Mean option score in male and female psoriasis patients by age groups. The Option Score represents the question “Does a medical person at your usual source of care present and explain all options to you?” Scores range from 1 through 4, with 4 representing the most favorable patient rating. The line represents the mean score, and the shaded areas represent the 95% Confidence Intervals.

We conducted a multivariate linear regression between the Option Score, representing patients’ evaluation of their clinicians’ explanation of all treatment options, and patient age in female psoriasis patients compared to male psoriasis patients, adjusting for covariates. Compared to age-matched male psoriasis patients, older female psoriasis patients demonstrated significantly higher Option Scores at ages 40–49 (female patient mean **3.91** [95% CI: 3.88–3.94] vs. male patient mean **3.80** [95% CI: 3.73–3.86], p<0.005), 50–59 (female patient mean **3.94** [95% CI: 3.91–3.96] vs. male patient mean **3.82** [95% CI: 3.77–3.87], p<0.005), 60–69 (female patient mean **3.97** [95% CI: 3.93–4.01] vs. male patient mean **3.84** [95% CI: 3.80–3.88], p<0.005), and >70 (female patient mean **4.00** [95% CI: 3.94–4.06] vs. male patient mean **3.87** [95% CI: 3.81–3.92], p<0.005). No significant differences were demonstrated in other age groups between male and female psoriasis patients (Fig 4).

### Analysis of patient education score by age and sex

**Fig 5:**
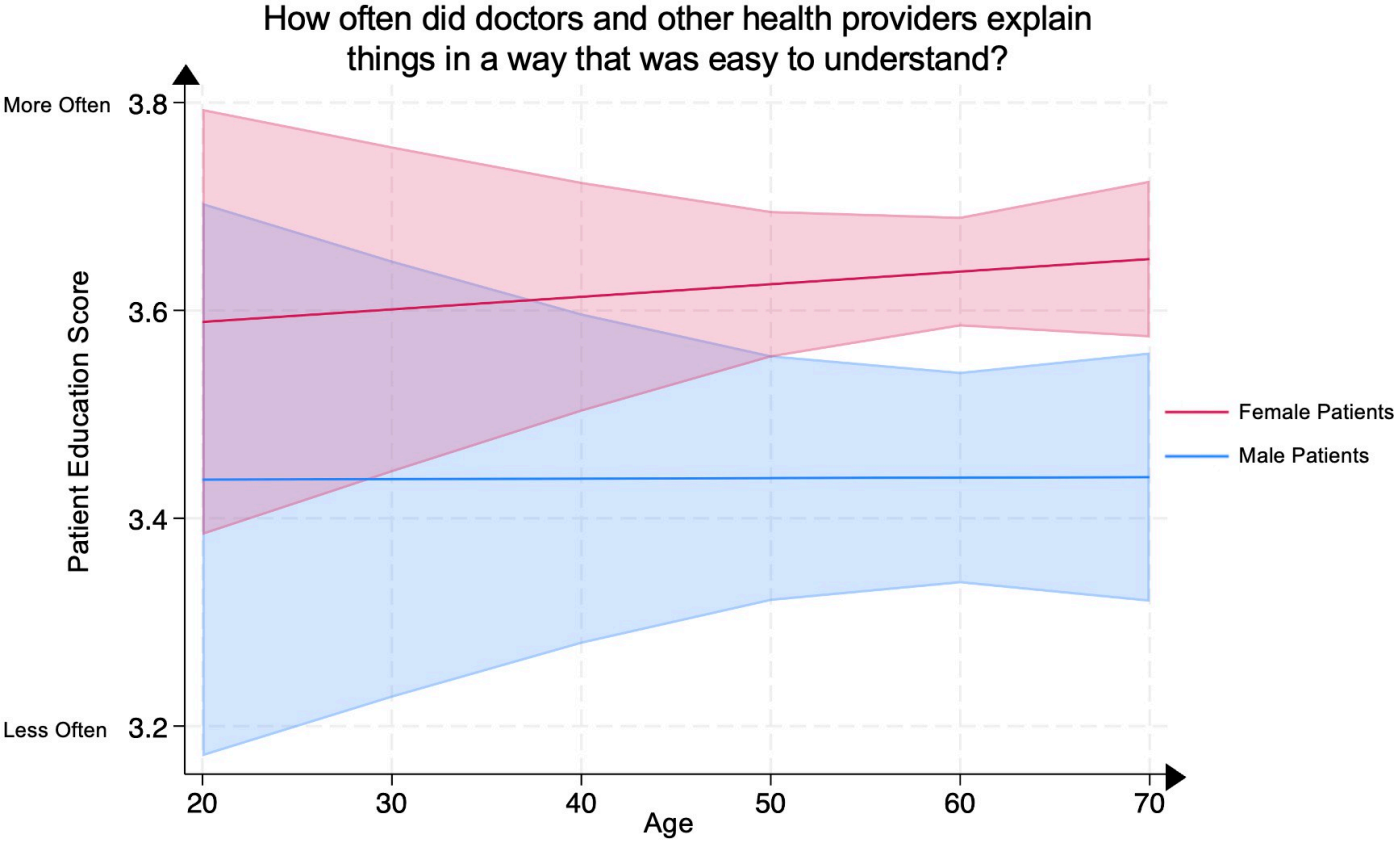
Mean patient education score in male and female psoriasis patients by age group. The Patient Education Score represents the question “How often did doctors and other health providers explain things in a way that was easy to understand?” Scores range from 1 through 4, with 4 representing the most favorable patient rating. The line represents the mean score, and the shaded areas represent the 95% Confidence Intervals.

We conducted a multivariate linear regression between the Patient Education Score, representing patients’ evaluation of their clinicians’ presentation of patient education in easily understood language, and patient age in female psoriasis patients compared to male psoriasis patients, adjusting for covariates. Compared to age-matched male psoriasis patients, older female psoriasis patients demonstrated significantly higher Patient Education Scores at ages 60–69 (female patient mean **3.64** [95% CI: 3.58–3.69] vs. male patient mean **3.44** [95% CI: 3.34–3.54], p<0.005) and >70 (female patient mean **3.65** [95% CI: 3.57–3.72] vs. male patient mean **3.44** [95% CI: 3.32–3.56], p<0.005). No significant differences were demonstrated in other age groups between male and female psoriasis patients (Fig 5).

### Analysis of listen, respect, and time spent scores by age and sex

**Fig 6:**
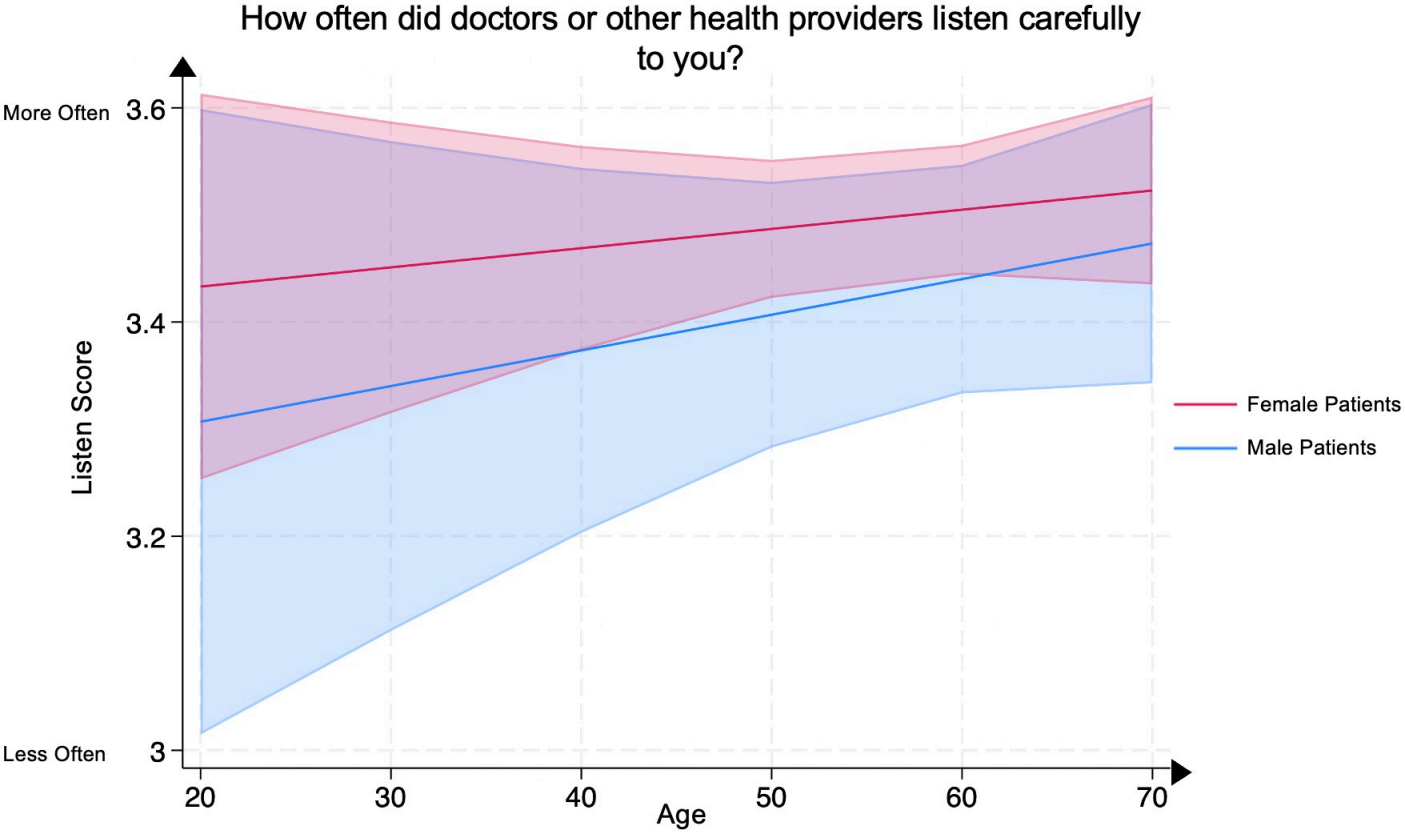
Mean listen score in male and female psoriasis patients by age groups. The Listen Score represents the question “How often did doctors or other health providers listen carefully to you?” Scores range from 1 through 4, with 4 representing the most favorable patient rating. The line represents the mean score, and the shaded areas represent the 95% Confidence Intervals.

**Fig 7:**
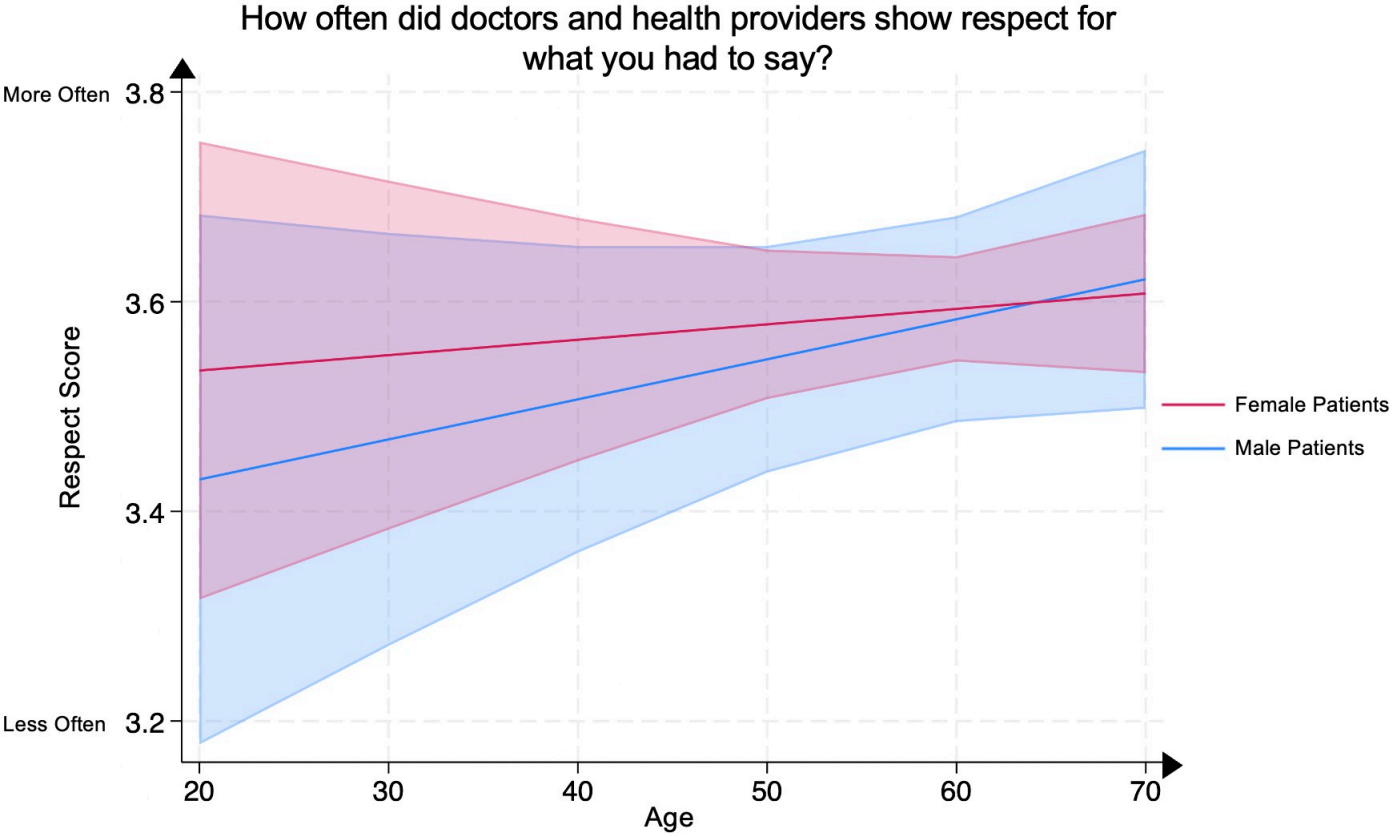
Mean respect score in male and female psoriasis patients by age group. The Respect Score represents the question “How often did doctors and health providers show respect for what you had to say?” Scores range from 1 through 4, with 4 representing the most favorable patient rating. The line represents the mean score, and the shaded areas represent the 95% Confidence Intervals.

**Fig 8:**
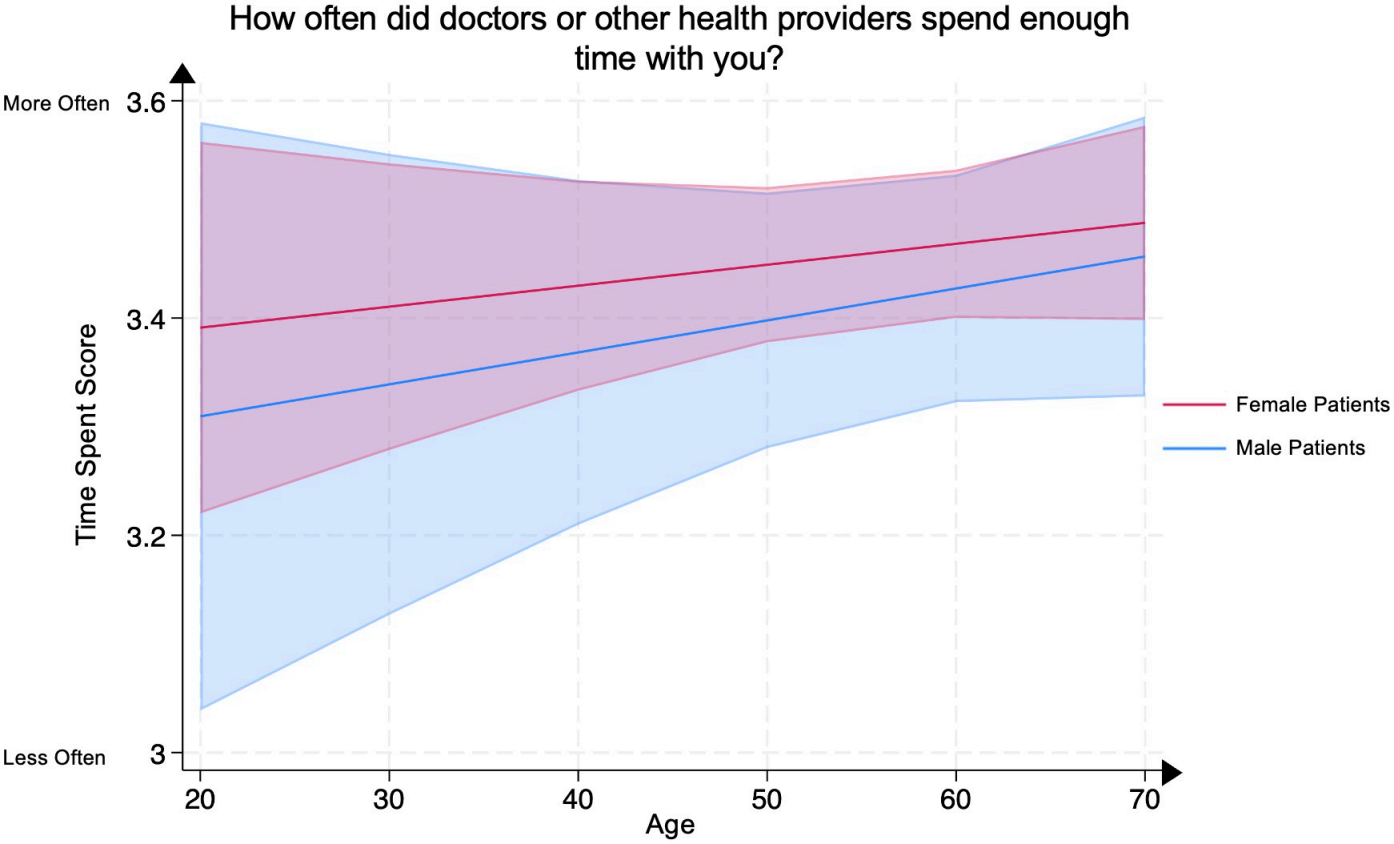
Mean time spent score in male and female psoriasis patients by age group. The Time Spent Score represents the question “How often did doctors or other health providers spend enough time with you?” Scores range from 1 through 4, with 4 representing the most favorable patient rating. The line represents the mean score, and the shaded areas represent the 95% Confidence Intervals.

On multivariate linear regression, no significant differences were demonstrated in any age group between male and female psoriasis patients for the (1) Listen Score, representing patients’ evaluation of their clinicians’ active listening (Fig 6), (2) Respect Score, representing patients’ evaluation of their clinicians’ respect for them (Fig 7), or (3) Time Spent Score, representing patients’ evaluation of the amount of time their clinicians spent with them (Fig 8). All analyses were adjusted for covariates.

### Analysis of composite SDM score by age

**Fig 9:**
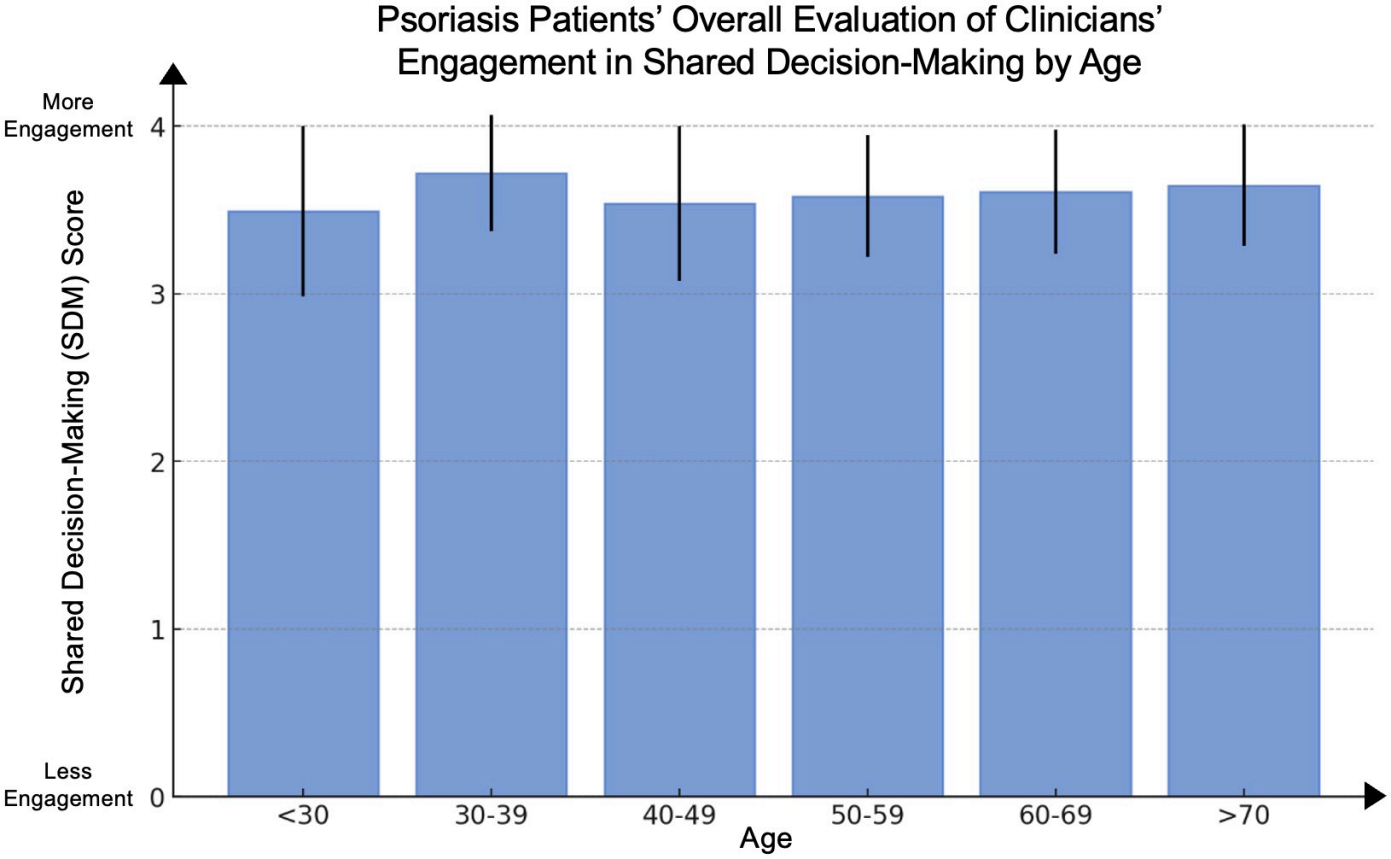
Psoriasis patients’ overall evaluation of clinicians’ engagement in shared decision-making by age. Mean composite SDM Score, representing patients’ overall evaluation of their clinicians’ engagement in SDM, in psoriasis patients by their age groups. Scores range from 1 through 4, with 4 representing the most favorable patient rating.

We also assessed differences in patients’ evaluations of clinicians’ engagement in SDM by patient age, independent of patient sex. We conducted a multivariate linear regression to compare the differences between patients’ evaluations of clinicians’ engagement in SDM across patient age groups, adjusting for covariates. There were no significant differences between patient age groups compared to psoriasis patients ages <30 (Fig 9).

## Discussion

SDM is crucial to formulating sustainable, patient-centered treatment plans. This is particularly important in psoriasis care given the multitude of available treatments and the high rates of patient non-adherence to their medical regimens. Our study is among the initial efforts to examine the effects of psoriasis patients’ age and sex on their evaluation of their clinicians’ engagement in SDM.

We found that older (>60 years old) female psoriasis patients evaluated their clinicians’ engagement in SDM significantly more favorably than their age-matched male counterparts. In particular, when evaluating the sub-domains of the composite SDM Score, we observed that older female psoriasis patients evaluated their clinicians significantly more favorably compared to their male counterparts regarding their clinician’s ability to (1) present and explain available treatment options (Option and Patient Education Scores), (2) ask the patient to help make their treatment decision (Decide Score), and (3) evaluate the patient’s medical history (History Taking Score). These SDM factors are integral to a physician’s ability to provide patients with individualized treatments that align with their medical needs and personal values.

In part, these results may be explained by findings from studies that have reported male patients to be significantly less likely to participate in shared decision-making with their providers [11], feel informed about their care [11], and prefer an active role in their health decision-making [17] compared to female patients. As male patients have been found to demonstrate lower engagement in their healthcare, a reciprocal dynamic may exist in which they perceive their clinicians to be less engaged with them. Additionally, our findings may be explained by prior studies’ reports that older patients are significantly less likely to challenge physicians or ask questions and more likely to delegate or defer decision-making [18–21]. The effects of male sex and older age may be synergistic and may explain the differences in older male and female psoriasis patients’ perceptions of their clinicians’ engagement in SDM that are not observed between younger male and female psoriasis patients. Our findings highlight the need for clinicians to adjust their delivery of SDM based on the demographic factors of each patient, as their preferences for care and communication may vary based on these identities.

In addition to age and sex differences, the mean SDM Score for our population of psoriasis patients was 3.6 out of 4. As prior literature has established an SDM Score of 3.8 or lower to represent an unfavorable evaluation of a patient’s perception of SDM [14, 16], our study highlights the need for overall improvement of SDM for all psoriasis patients. One strategy to improve clinicians’ delivery of SDM is visual aids, which have been found to improve patient knowledge and engagement in the decision-making process for biologic counseling in the dermatology setting [22]. These patient education tools may help improve all patients’ evaluations of the decision-making process.

Our findings should be interpreted within the context of the study’s design. Patients’ perceptions of their clinicians’ delivery of SDM may be influenced by a number of factors. While we adjusted for identifiable confounding factors in the MEPS, such as patients’ comorbidities, socioeconomic factors, and disease severity, there may be other factors that were not captured in the database, such as individual preferences, previous experiences, and cultural beliefs. Therefore, future qualitative studies that also account for these additional factors will be important.

## Conclusion

We evaluated the effect of psoriasis patients’ ages and sex on their evaluation of their clinicians’ engagement in SDM. We found that older female psoriasis patients perceived their clinicians to be significantly more engaged in shared decision-making as compared to age-matched male psoriasis patients. These differences were most pronounced when evaluating a clinician’s ability to present and explain available treatment options, ask the patient to help make their treatment decision, and evaluate what modalities best suit the patient’s medical history. Therefore, it is important for clinicians to consider how patients’ demographic factors may affect their perception of SDM. Further inquiry into patient- and physician-reported factors affecting the delivery of SDM will allow clinicians to improve communication and engagement with patients in the development of psoriasis treatment plans.
